# Polydeoxyribonucleotide in Female Pattern Hair Loss: Current Evidence and Knowledge Gaps

**DOI:** 10.1111/jocd.71028

**Published:** 2026-06-30

**Authors:** Nika Bakshi, Andrea Dai, Elham T. Tabatabaei, Steven Daveluy

**Affiliations:** ^1^ Department of Dermatology Wayne State University School of Medicine Detroit Michigan USA; ^2^ Department of Dermatology Henry Ford Health System Detroit Michigan USA

Ten years ago, a study by Lee et al. reported polydeoxyribonucleotide (PDRN), a mixture of deoxyribonucleotide polymers, as a promising therapeutic option for female pattern hair loss. The randomized controlled trial showed meaningful improvements in both hair density and thickness, suggesting a potential role for PDRN as a regenerative treatment in alopecia [1]. However, these findings were derived from a small sample size of a single study and require validation before they can be considered for clinical practice. This commentary revisits Lee et al.'s work in the context of growing interest in regenerative injectables and highlights the importance of further studies to confirm reproducibility before clinical application can be contemplated.

## 1 Mechanism of Action and Regenerative Profile

PDRN consists of low molecular weight deoxyribonucleotides derived from *Oncorhynchus mykiss* (Salmon trout) or *Oncorhynchus keta* (Chum Salmon) [2]. Along with its longer‐chain counterpart, polynucleotide (PN), PDRN has gained increased visibility in aesthetic medicine, particularly in East Asian dermatologic practice. Most commonly, it is delivered via intradermal or subdermal injections to improve skin hydration, texture, and overall dermal quality [3].

Recently, PDRN has been grouped with a broader class including platelet‐rich plasma (PRP), exosomes, and stem‐cell‐derived vesicles that act by promoting angiogenesis, growth factor signaling, and fibroblast activation [4]. Unlike PRP, whose composition varies based on patient physiology and preparation technique, PDRN is a chemically defined and consistently manufactured product.

The biologic plausibility for PDRN in female pattern hair loss stems from its capacity to address two key pathophysiologic features: microvascular dysfunction and chronic low‐grade inflammation [5]. By activating the adenosine A2A receptor, PDRN upregulates vascular endothelial growth factor (VEGF), driving neovascularization and enhancing oxygen and nutrient delivery to the dermal papilla [2]. Additionally, PDRN downregulates proinflammatory mediators such as TNF‐α and IL‐6, reducing perifollicular inflammation and supporting a more favorable environment for anagen‐phase maintenance [6, 7]. Together, these mechanisms provide a biologic rationale for its use in hair restoration.

Polynucleotides (PNs), which are structurally related to PDRN but consist of longer DNA fragments, have also been explored in regenerative dermatology [8]. In contrast to PDRN, which primarily exerts its effects through adenosine A2A receptor–mediated signaling, PNs are thought to provide a more structural, scaffold‐like role in tissue repair [2, 8]. While PNs have been studied in aesthetic and wound‐healing contexts, there are no randomized controlled trials evaluating their efficacy in female pattern hair loss. As such, whether PNs offer any clinical advantage over PDRN in hair follicle biology remains unclear.

## 2 Clinical Efficacy and Safety in Female Pattern Hair Loss

In the only randomized controlled trial of 40 women with female pattern hair loss, participants received weekly intra‐perifollicular injections of either PDRN alone or combined with PRP [1]. After 12 weeks, the PDRN‐only group demonstrated a statistically significant improvement in mean hair count (17.9% ± 13.2%) and mean hair thickness (13.5% ± 10.7%) [1], providing the first clinical evidence supporting PDRN as a stand‐alone agent for hair growth.

While Lee et al. provided the first randomized trial evaluating PDRN in female pattern hair loss, several limitations restrict interpretation. The lack of documentation regarding randomization and allocation concealment procedures makes it difficult to rule out selection bias. Moreover, since the trial compared PDRN monotherapy against a combination of PDRN and PRP, it is difficult to determine the true efficacy of the therapy. The absence of a saline or sham control group does not rule out a potential placebo response.

There is also an issue regarding reproducibility across clinical settings. The injection protocol was described as 2 mL of PDRN along the frontal, mid, and vertex areas for 12 sessions. However, the study does not specify whether procedures were performed by a single clinician. There is also no mention of standardization of injection technique, which can impact reproducibility.

Regarding outcome assessment, a computerized phototrichogram analysis of hair counts and thickness was conducted by a blinded evaluator. However, details regarding how blinding was maintained across sessions are not fully described. Although statistically significant improvements in hair count and thickness were observed, there is no report of patient‐centered outcomes or physician assessments, making it difficult to understand the clinical significance of these findings. In addition, rates of loss to follow‐up and the use of intention‐to‐treat analysis are not reported, making it difficult to exclude potential attrition bias.

Given these limitations, the findings should be interpreted as exploratory.

## 3 Combination Therapy and Biologic Complementarity

The addition of PRP to the PDRN regimen resulted in a further increase in hair thickness (*p* = 0.031), but did not significantly affect hair count (*p* > 0.05) [1]. While PRP delivers a transient boost of growth factors such as PDGF, VEGF, and IGF‐1 that promote follicular proliferation, PDRN provides sustained angiogenic and anti‐inflammatory signaling through continuous A2A receptor activation [1, 9]. For PRP, the release of growth factors occurs within minutes to hours and has been studied in human in vivo studies [10]. In contrast, PDRN's effects occur through receptor signaling, with activity observed several days to weeks [2, 6]. These conclusions are drawn from pharmacologic studies rather than human trials, and there is no data demonstrating that these pathways are activated within the hair follicle [2, 6]. As such, whether these mechanisms support clinically meaningful hair growth remains uncertain. These observations suggest a potential role for PDRN as a complementary agent in regenerative therapies, though further clinical validation is required.

## 4 Safety and Tolerability Profile

Across both regenerative and aesthetic applications, PDRN has demonstrated a favorable safety profile. The compound undergoes high‐temperature purification to achieve over 95% nucleotide purity, minimizing immune reactivity from residual proteins [11]. Toxicology studies show no systemic or organ toxicity with repeated administration in animal models [2]. Clinically, PDRN‐based fillers have been well tolerated, with only mild, transient reactions such as erythema or swelling reported [11]. Taken together, these findings support PDRN's safety as a cutaneous injectable.

## 5 Research Gap and Future Directions

Despite the initial outcomes reported by Lee et al., there has been minimal follow‐up research on PDRN for alopecia over the past decade. No additional randomized controlled trials have been published, leaving clinicians with almost no evidence to guide practice. A non‐systematic search of PubMed up to October 2025 identified no additional randomized controlled trials evaluating PDRN in female pattern hair loss beyond Lee et al. [1], although a limited number of small case series and combination therapy studies have been reported. In addition, structurally related compounds such as polynucleotides (PNs) have also been explored in regenerative dermatology, though comparative data between PNs and PDRN in hair disorders remain limited [12].

This leaves the evidence base limited to a single study without subsequent replication. Several factors may have contributed to this gap. PDRN entered aesthetic dermatology largely through off‐label cosmetics use, independent of major pharmaceutical investment, resulting in fewer formal trials. Additionally, commercial and research efforts have concentrated more heavily on PRP, stem‐cell‐derived therapies, and exosomes, overshadowing PDRN despite its accessible manufacturing and reported safety profile.

Future research should focus on adequately powered randomized controlled trials. As an illustrative example, a study enrolling approximately 100–120 participants may provide greater precision and generalizability than the existing 40‐patient trial, although formal sample size calculations should be based on the expected treatment effect and primary outcome measures. Such studies should incorporate a sham or saline injection control group as well as a topical minoxidil standard‐of‐care comparator, with blinded outcome assessment. A treatment duration of at least 24 weeks would be important to assess the durability of response. Primary outcomes should be assessed utilizing both objective methods, such as validated phototrichogram techniques, and patient‐centered outcomes and physician assessments. If a randomized controlled trial is not feasible, prospective registries or real‐world observational studies may provide complementary data.

Female pattern hair loss affects millions of women worldwide and substantially impacts quality of life and psychological well‐being. Given this burden, patients can inquire about emerging therapies such as PDRN, and clinicians should counsel that current evidence is limited and that clinical benefit remains uncertain. PDRN should be considered investigational, and its use should be approached with caution until further data is available.

## Author Contributions

N.B. and A.D. drafted the manuscript and completed the literature review. E.T.T. and S.D. provided critical revision and subject‐matter expertise. All authors reviewed and approved the final manuscript. All authors agree to be accountable for all aspects of the work in ensuring that questions related to the accuracy/integrity of the work are investigated and resolved.

## Ethics Statement

The authors have nothing to report.

## Conflicts of Interest

S.D. reports serving as a speaker and consultant for AbbVie, UCB, and Novartis; as an investigator for Pfizer, UCB, AbbVie, Sanofi, Regeneron, Novartis, MoonLake, Insmed, and Incyte; and as a consultant for Incyte. The remaining authors declare no conflicts of interest.

## Data Availability

Data sharing not applicable to this article as no datasets were generated or analysed during the current study.
